# Physapubescin selectively induces apoptosis in VHL-null renal cell carcinoma cells through down-regulation of HIF-2α and inhibits tumor growth

**DOI:** 10.1038/srep32582

**Published:** 2016-09-01

**Authors:** Lixia Chen, Guiyang Xia, Feng Qiu, Chunli Wu, Andria P. Denmon, Xiaolin Zi

**Affiliations:** 1 Departments of Urology and Pharmacology, Chao Family Comprehensive Cancer Center, University of California, Irvine, Orange, CA 92868, USA; 2 Department of Natural Products Chemistry, School of Traditional Chinese Materia Medica, Key Laboratory of Structure-Based Drug Design & Discovery, Ministry of Education, Shenyang Pharmaceutical University, Shenyang 110016, P. R. China; 3 School of Chinese Materia Medica and Tianjin State Key Laboratory of Modern Chinese Medicine, Tianjin University of Traditional Chinese Medicine, Tianjin 300193, P. R. China; 4 Co-innovation Center of Henan Province for New Drug R & D and Preclinical Safety and School of Pharmaceutical Sciences, Zhengzhou University, 100 Kexue Avenue, Zhengzhou, Henan 450001, P. R. China

## Abstract

We have purified physapubescin, a predominant steroidal lactone, from medicinal plant *Physalis pubescens* L., commonly named as “hairy groundcherry” in English and “Deng-Long-Cao” in Chinese. Von Hippel-Lindau (VHL)-null 786-O, RCC4 and A498 Renal Cell Carcinoma (RCC) cell lines expressing high levels of Hypoxia Inducible Factor (HIF)-2α are more sensitive to physapubescin-mediated apoptosis and growth inhibitory effect than VHL wild-type Caki-2 and ACHN RCC cell lines. Restoration of VHL in RCC4 cells attenuated the growth inhibitory effect of physapubescin. Physapubescin decreases the expression of HIF-2α and increases the expression of CCAAT/enhancer-binding protein homologus protein (CHOP), which leads to up-regulation of death receptor 5 (DR5), activation of caspase-8 and -3, cleavage of poly (ADP-Ribose) polymerase (PARP) and apoptosis. Under hypoxia conditions, the apoptotic and growth inhibitory effects of physapubescin are further enhanced. Additionally, physapubescin synergizes with TNF-related apoptosis-inducing ligand (TRAIL) for markedly enhanced induction of apoptosis in VHL-null 786-O cells but not in VHL wild-type Caki-2 cells. Physapubescin significantly inhibited *in vivo* angiogenesis in the 786-O xenograft. Physapubescin as a novel agent for elimination of VHL-null RCC cells via apoptosis is warranted for further investigation.

*Physalis pubescens* L. (Solanaceae) is a flowering plant that produces nutritious and healthy fruit, commonly known as husk tomato and hairy groundcherry in English; muyaca and capulí in Spanish; and Deng-Long-Cao in Chinese^1^,^2^. *Physalis pubescens* L. has been used in traditional folk medicine for the treatment of sore throat, cough, and urogenital system diseases such as urethritis, hematuria, orchitis^1^,^2^. We therefore have carried out a phytochemical study on this plant and identified several withanolides from this plant. Physapubescin is the most abundant withanolide that constitutes up to 0.033% dry weight of the hairy groundcherry.

Withanolides are a group of polyoxygenated C_28_-ergostane lactones or lactols that have attracted significant research interest as a new class of anti-cancer agents due to their diversified chemical structures as well as their antitumor^3^,^4^,^5^,^6^, anti-inflammatory^3^,^7^, immunomodulating activities^3^,^8^ to name a few. Since the first withanolide-type compound, withaferin A, was isolated from *Withania somnifera* in 1965^9^, more than 750 withanolides with diversified functional groups have been identified from the Solanaceae family^10^. These withanolides can be divided into more than 22 structure types, such as normal withanolides, physalins, jaborols, acnistins, withajardins, neophysalins, *etc.*^3^. The structure-activity relationship analyses of these withanolides suggested that a Δ^2^−1-oxo-functionality in ring A and a 5β, 6β-epoxy in ring B were required for their cytotoxic activity^3^,^10^. Among these withanolides, the anti-cancer mechanisms of withaferin A have been extensively studied and a key structural property, α, β-unsaturated ketone moiety in the A ring, identified. The α, β-unsaturated carbonyl group of withaferin A directly binds to cysteine thiols of several key signaling proteins, such as Vimentin^11^, Glial fibrillary acidic protein^12^, IκB kinase β^13^ and β-Tubulin^14^, leading to modulation of important cancer pathways, such as cell survival, apoptosis, angiogenesis, stress response, inflammation and immune regulation. In contrast to withaferin A and other withanolides (e. g. withanolide E, physalins, and tubocapsanolide A)^15^,^16^,^17^,^18^, the potential of physapubescin as a new anti-cancer agent remains largely unexplored.

Von Hippel-Lindau (VHL) protein is a member of an E3-ubiquitin ligase complex that target α subunits of hypoxia-inducible factors 1 and 2 (HIFα) for their ubiquitin-mediated, proteasomal degradation^19^. The loss of VHL through somatic mutation or hypermethylation occurs in over 80% of clear cell Renal Cell Carcinoma (ccRCC) and is the main driver for cancer growth and metastasis^20^. VHL inactivation leads to HIF-α stabilization and nuclear translocation^19^. In the nucleus, HIF-1 and -2α act as transcription factors to stimulate expression of genes that are involved in angiogenesis, anaerobic metabolism, cell proliferation, and survival^19^. HIF-2α has been shown to be an oncogene and required for tumor growth in xenograft models^21^,^22^,^23^,^24^. In contrast, ectopic overexpression of HIF-1α suppresses xenograft tumor growth^21^,^22^,^23^,^24^. HIF-1α and HIF-2α direct distinct transcriptional profiles^25^. Therefore, inhibition of HIF-2α expression and/or function would be an important approach for developing novel agents in treatment of ccRCC with loss of VHL.

Here, we have shown that physapubescin, at concentrations of 2.5 μM and 5 μM, selectively induces apoptosis in 786-O, RCC4 and A498 VHL-null RCC cell lines, but it has a minimal apoptotic effect on the wild-type Caki-2 RCC cell line. Physapubescin decreases the protein levels of HIF-2α and increases the expression of CCAAT/enhancer-binding protein homologous protein (CHOP), which result in an increased expression of death receptor 5 (DR5) and activation of caspases-8 and -3 to induce apoptosis. The apoptotic effect of physapubescin is further enhanced by the addition of TNF-related apoptosis-inducing ligand (TRAIL) or under hypoxic conditions. Furthermore, physapubescin demonstrates significant *in vivo* anti-angiogenesis activities in the 786-O xenograft model.

## Results

### Physapubescin preferentially inhibits the growth of VHL-null RCC cells

**Figure 1:**
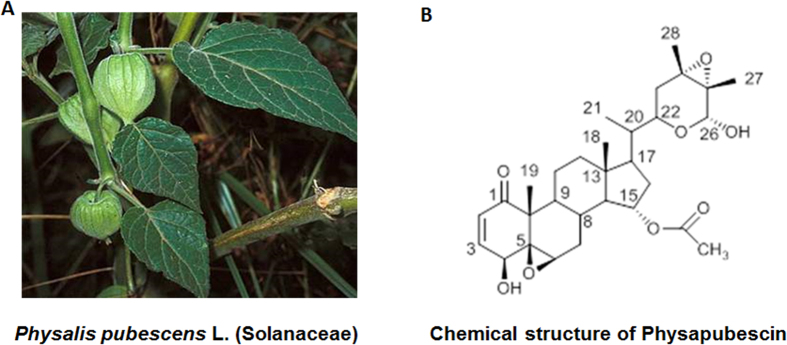
Photograph of *Physalis pubescens* L. and the chemical structure of physapubescin.

Physapubescin was isolated from *Physalis pubescens* L. extracts and its chemical structure was identified by comparing its nuclear magnetic resonance (NMR) spectroscopic data with those of the published values (supplementary Table 1, supplementary Fig. 1A,B). The purity of physapubescin was determined by High Performance Liquid Chromatography (HPLC) to be 98.1% (supplementary Fig. 2 and Fig.1) and used in all the experiments.

**Figure 2:**
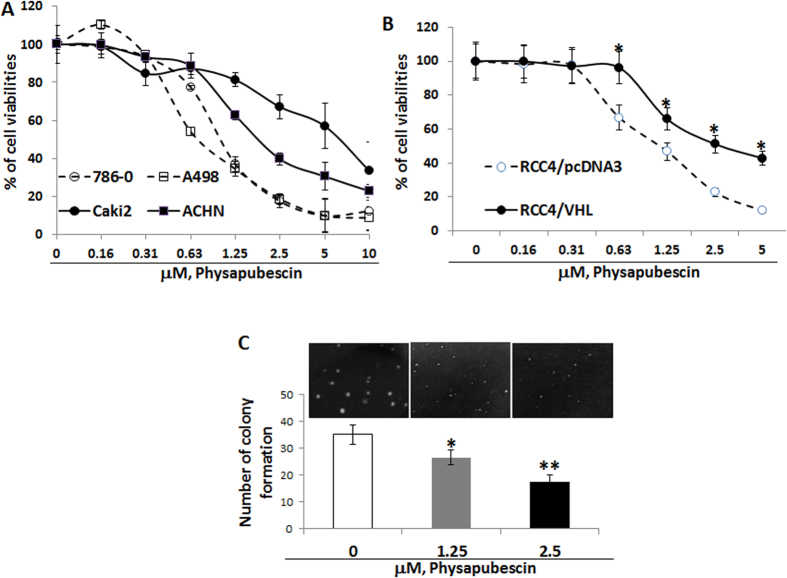
Physapubescin inhibits the anchorage-dependent and independent growth of RCC cell lines. (**A,B**) cells in 24-well culture plates were treated with 0.05% DMSO or physapubescin at the indicated concentrations. After 72 hours of treatment, cell densities were measured by MTT assay. Each point is the mean of values from four independent plates; bars, SEM. Each sample was counted in duplicate. (**C**) a soft agar colony formation assay with indicted treatments of 786-O cells was performed using 6- well plates. The data are presented by bar figure and mean ± SEM of four independent wells at optimum time of 21 days post cell seeding; Pictures are qualitative analysis of colony formation under an inverted microscope. “*” and “**” denote “*P* < 0.05” and “*P* < 0.01” respectively.

In Fig. 2A, physapubescin inhibits the growth of RCC cell lines (786-O, A-498, Caki-2 and ACHN) in a dose-dependent manner. The effect of physapubescin on the growth of RCC cells is expressed as percentage of cell viability relative to control. The IC_50s_ of physapubescin for 786-O, A-498, ACHN and Caki-2 cells are estimated to be ∼1.08 μM, 1.06 μM, 2.25 μM and 5.5 μM, respectively (Fig. 2B). Both 786-O and A-498 cells harbor a VHL deletion mutation and express high levels of HIF-2α protein, but no HIF-1α protein^26^. 786-O and A-498 cells are two to five times more sensitive to the treatment of physapubescin than Caki-2 and ACHN cells, which express wild-type VHL (Fig. 2A, *Ps* < 0.05).

Figure 2B shows that RCC4/VHL cells (a subline stably overexpressing wild-type pVHL) are about 2.5 times less sensitive to the growth inhibitory effect of physapubescin than RCC4 cells, which are VHL deficient^27^, were transfected with vector control pcDNA3 (IC_50s_ for RCC4/VHL *vs* RCC4/pcDNA3 cells were estimated to be 2.5 ± 0.14 μM *vs* 1.02 ± 0.08 μM, *P* < 0.01). This result suggests that re-expression of wild-type VHL attenuates the growth inhibitory effect of physapubescin.

Figure 2C demonstrates that physapubescin, at concentrations of 1.25 μM and 2.5 μM, significantly reduces the colony formation of 786-O cells in soft agar by ~25 and ~50%, respectively, compared to vehicle control treatment [0.05% dimethyl sulfoxide (DMSO)] (*Ps* < 0.05).

### Physapubescin selectively induces apoptosis in VHL-null 786-O RCC cells, but not in VHL wild-type Caki-2 cells

**Figure 3:**
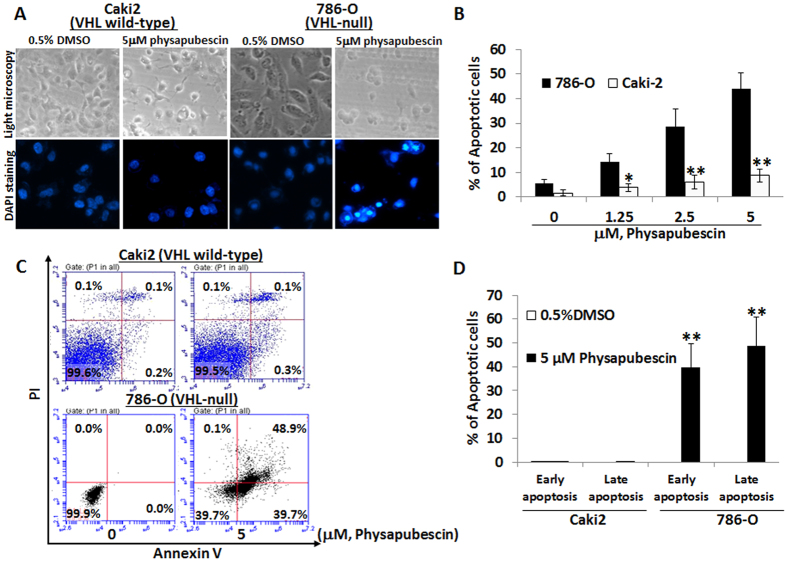
Physapubescin selectively induces apoptosis in VHL-null 786-O cells but not VHL wild-type Caki-2 cells. (**A**) the upper panel: live cell morphology under phase-contrast light microscope (Magnification: X100). DAPI staining of nuclear morphology under fluorescence microscope (Magnification: X200). Representative pictures were shown from a random field. (**B**) cells with nuclear condensation and fragmentation were counted as apoptotic cells in 12 fields in each group. The percentage of apoptotic cells was calculated and presented as mean ± SEM. (**C,D**) cells were stained by Annexin V and PI and analyzed by flow cytometry. The data are presented by bar figure and mean ± SEM of three independent experiments. “*” and “**” denote “*P* < 0.05” and “*P* < 0.01” respectively.

We next determined whether the differential growth inhibitory effects on VHL-null *vs.* wild-type cells by physapubescin are associated with their sensitivity to apoptosis induction. Apoptotic morphology of control- and physapubescin-treated cells was examined under light and fluorescence microscopes (Fig. 3A). After 4′, 6-diamidino-2-phenylindole (DAPI) nuclear staining, cells with nuclear fragmentation and condensation were counted as apoptotic cells. Figure 3B demonstrates that physapubescin treatment of VHL-null 786-O cells at concentrations of 1.25 μM, 2.5 μM and 5 μM for 24 hours resulted in 14.2 to 44.1% of cells undergoing apoptosis in a dose-dependent manner, whereas vehicle control (0.05% DMSO) treatment resulted in ~5.7% increase over the background of apoptotic cells (*Ps* < 0.01). The same concentrations were applied with physapubescin and there was a minimal apoptotic effect on VHL wild-type Caki-2 cells. Percentages of apoptotic cells after physapubescin treatment of Caki-2 cells ranged from 3.8 to 8.7% (Fig. 3B).

Flowcytometry analysis of Annexin V and Propidium iodide (PI) stained cells confirmed that physapubescin treatment of VHL-null 786-O cells at concentrations of 2.5 μM and 5 μM resulted in a significant increase in both early (Annexin V staining only, right-lower panels) and late apoptosis (Annexin V and PI staining, right-upper panels) populations as compared to control treatment (Fig. 3C,D, 20.60 ± 5.0% early apoptotic cells and 71.70 ± 5.81% late apoptotic cells in 5 μM physapubescin treated 786-O cells, respectively, vs. 0.00 ± 0.00% early apoptotic cells and 0.03 ± 0.06% late apoptotic cells in control treated 786-O cells; Student’s t test, *Ps* < 0.01). Physapubescin at the same concentrations induced minimal apoptosis in VHL wild-type Caki-2 cells (Fig. 3C,D).

### Physapubescin results in cleavage of caspase-8/3 and poly (ADP-ribose) polymerase (PARP) via down-regulation of HIF-2α and up-regulation of CHOP and DR5 protein expression in 786-O but not in Caki-2 cells

**Figure 4:**
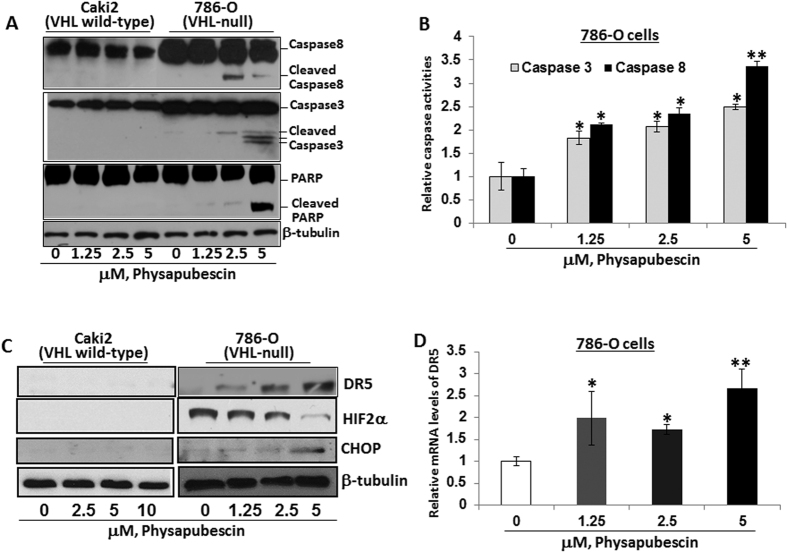
Physapubescin decreases the expression of HIF-2α and increased the expression of CHOP and DR5 leading to activation of caspase cascade in 786-O cells but not Caki-2 cells. (**A**) cleaved caspase-8, -3 and PARP after indicated treatments for 24 hours was detected by Western blotting analysis. β-Tubulin was detected as a loading control. A representative blot was shown from three independent experiments. (**B**) caspase activation was determined with a caspase-3/7 or caspase-8 activity assay. Bars are means ± SEMs of three independent quantitative measures. (**C**) the protein expression of DR5, HIF-2α and CHOP after indicated treatments for 24 hours was analyzed by Western blotting analysis. A representative blot was shown from three independent experiments. (**D**) Quantitative RT-PCR analysis of DR5 mRNA expression. Bars are means ± SEMs of three independent quantitative measures. “*” and “**” denote “*P* < 0.05” and “*P* < 0.01” respectively.

To see whether the apoptotic effect of physapubescin is involved in activation of a cascade of caspases, the cleavage of caspase-8, caspase-3, and PARP were detected by Western blotting and the activities of caspase-8 and -3 were measured by ELISA. Physapubescin treatment at 2.5 μM and 5 μM concentrations caused activation of caspase-8 and caspase-3, as well as cleavage of PARP in a dose-dependent manner in 786-O cells, but with minimal effects on Caki-2 cells (Fig. 4A,B).

Activation of caspase-8 is associated with the extrinsic apoptotic pathway via death receptor activation^4^,^5^. Figure 4C shows that treatment of 786-O cells with physapubescin at 1.25 μM to 5 μM concentrations caused a dose-dependent increase in the protein expression of DR5. This result is accompanied by increased expression of CHOP and decreased expression of HIF-2α. HIF-2α but not HIF-1α is highly expressed in some VHL-null RCC cell lines (e.g. 786-O and A-489)^26^. Similarly, physapubescin treatment of A-489 cells at 1.25 μM to 5 μM concentrations resulted in decreased expression of HIF-2α and increased expression of CHOP and DR5 leading to activation caspase-8 and PARP cleavage mediated apoptosis (Supplementary Fig. 3). Caki-2 cells did not express HIF-2α or exhibit induction of CHOP and DR5 expression (Fig. 4C). CHOP is a major transcriptional factor that regulates DR5 mRNA transcription^28^. The mRNA levels of DR5 were also significantly up-regulated by physapubescin (Fig. 4D).

### Physapubescin acts synergistically with TRAIL in reducing cell viability and inducing cleavage of caspase-3 and PARP in 786-O cells, but not in Caki-2 cells

**Figure 5:**
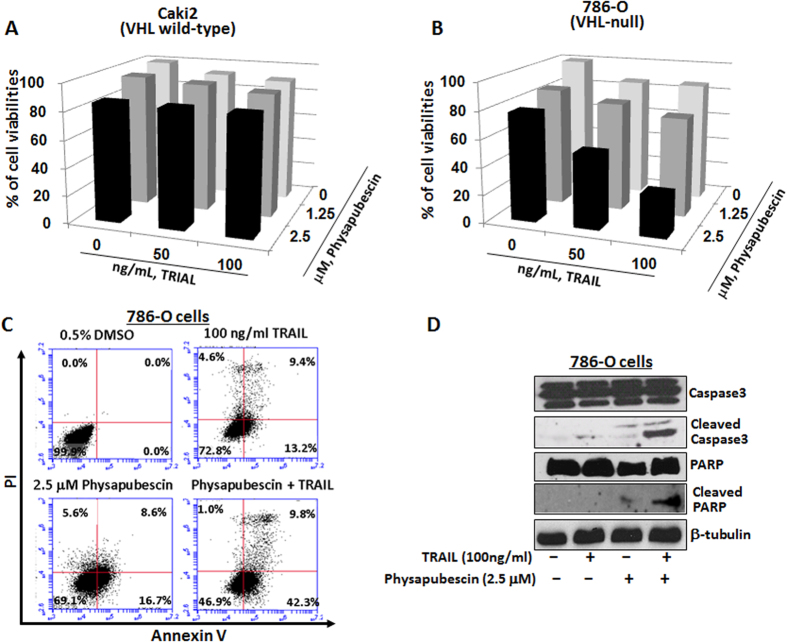
Physapubescin acts synergistically with TRAIL in VHL-null 786-O cells but not VHL wild-type Caki-2 cells. (**A,B**) the combined effect of physapubescin and TRAIL on 786-O and Caki-2 cell viability. Columns, mean for percentage of cell viability relative to control (n = 3); bars, SEM. (**C**) The combined effect of physaubescin and TRAIL on apoptosis of 786-O cells. Cells were stained by Annexin V and PI and analyzed by flow cytometry. Data represent the means from three independent experiments. SEMs are less than 5%. (**D**) the combined effects of physaubescin and TRAIL on caspase-3 and PARP activation after indicated treatments 24 hours were detected by Western blotting analysis. A representative blot was shown from three independent experiments.

In VHL wild-type Caki-2 cells, 2.5 μM physapubescin alone, TRAIL treatment alone, or their combinations, only caused minimal reduction of the cell viabilities by 7 to 15% (Fig. 5A).

However, in VHL-null 786-O cells, 2.5 μM physapubescin and 100 ng/ml TRAIL decreased the cell viabilities by 51.6% and 8.5%, respectively, whereas their combination led to a marked reduction of cell viability by 83% (Fig. 5B, *Ps* < 0.01).

Western blotting analysis further revealed that 2.5 μM physapubescin in combination with 100 ng/ml TRAIL for treatment of 786-O cells caused an enhanced cleavage of caspase-3 and PARP. Taken together, these results suggest that physapubescin may sensitize TRAIL in induction of apoptosis in VHL-null RCC cells (Fig. 5C).

### RCC cells under hypoxia conditions are more sensitive to physapubescin treatment in reducing cell viabilities and in expression of related biomarkers

**Figure 6:**
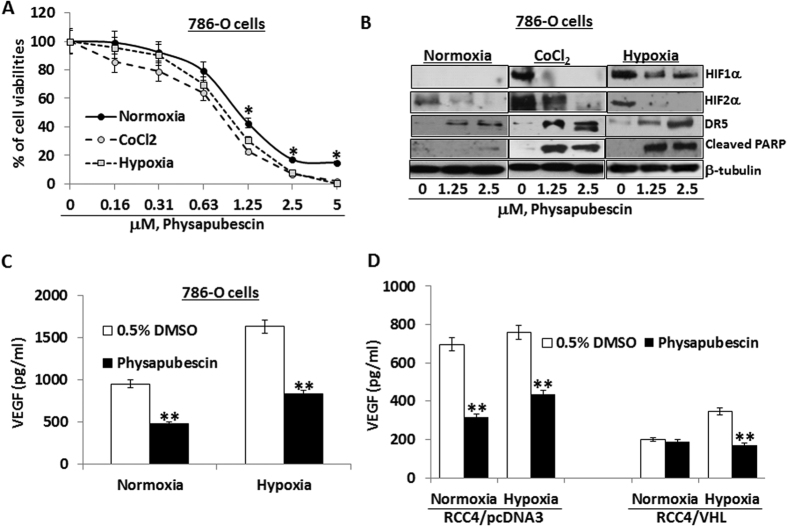
The effects of physapubescin on reducing cell viabilities, inducing apoptosis and modulating expression of related biomarkers are enhanced under hypoxia vs. normoxia conditions. 786-O cells were seeded at a density of 5 × 10^4^ cells/well in six well plates under normoxic (21% O_2_), hypoxic (1% O_2_) or hypoxia mimic (250 μM CoCl_2_) conditions. (**A**) After 24 hours of seeding, cells were treated with 0.05% DMSO or physapubescin at the indicated concentrations for 72 hours. Cell densities were measured by MTT assay. Each value represents mean ± SEM of three samples for each treatment. (**B**) After 24 hours of seeding, the protein expression of HIF-1α, HIF-2α, DR5, and cleaved PARP at indicated treatments for 24 hours was analyzed by Western blotting. β-Tubulin was detected as a loading control. A representative blot was shown from three independent experiments. (**C**) After 24 hours of seeding, the secretion of VEGF in conditioned medium at indicated treatments for 24 hours was analyzed by ELISA. Each value represents mean ± SEM of three samples for each treatment. (**D**) RCC4/pcDNA3 and RCC4/VHL cells were seeded at a density of 5 × 10^4^ cells/well in six well plates under normoxic (21% O_2_) or hypoxic (1% O_2_) conditions. After 24 hours of seeding, the secretion of VEGF in conditioned medium at indicated treatments for 24 hours was analyzed by ELISA. Each value represents mean ± SEM of three samples for each treatment. “*” and “**” denote “*P* < 0.05” and “*P* < 0.01” respectively.

Under hypoxia and hypoxia mimic [in presence of 250 μM cobalt chloride (CoCl_2_) conditions], the IC_50s_ of physapubescin for reducing cell viability of 786-O cells were estimated to be 0.81 ± 0.07 and 0.73 ± 0.10 μM, respectively, which were significantly lower compared to the value observed under normoxia condition (IC_50_ of physapubescin was 1.12 ± 0.12 μM) (Fig. 6A, *Ps* < 0.05). In particular, physapubescin at 1.25 μM, 2.5 μM and 5 μM concentrations were more effective under hypoxia and hypoxia mimic conditions than normoxia condition in reducing cell viabilities by 10 to 20% (Fig. 6A, *Ps* < 0.05). Similarly, RCC4 cells stably expressing vector control or VHL were more sensitive to the effect of physapubescin on reducing cell viabilities under hypoxia conditions compared to normxia (Supplementary Fig. 4A,B).

As expected, hypoxia and CoCl_2_ induced expression of HIF-1α and further enhanced the expression of HIF-2α (Fig. 6B). Physapubescin at concentrations of 2.5 μM and 5 μM markedly down-regulated the protein expression of both HIF-1α and HIF-2α, which was accompanied by enhanced induction of DR5 expression and PARP cleavage (Fig. 5B). The induction of DR5 and PARP cleavage by physapubescin was also enhanced in RCC4 cells stably expressing vector control or VHL under hypoxia conditions compared to normoxia condition (Supplementary Fig. 4C). These results indicate that physapubescin induces more apoptosis under hypoxia conditions by down-regulating HIF1/2α expression and upregulating DR5 expression.

Vascular endothelial growth factor (VEGF) is a major transcriptional target of both HIF-1α and HIF-2α^24^,^25^. We therefore analyzed the levels of VEGF secretion in the culture media of physapubescin treated 786-O cells. Figure 6C shows that hypoxia alone increased VEGF secretion. Interestingly, treatment with 5 μM physapubescin decreased VEGF secretion levels under normoxia and hypoxia conditions. At the same concentrations, in vector control transfected RCC4 cells, physapubescin also reduced VEGF secretion under normoxia and hypoxia conditions (Fig. 6D). In contrast, treatment of RCC4 cells stably overexpressing VHL with physapubescin did not affected VEGF secretion under normoxia condition, but it did significantly decreased VEGF secretion under hypoxia conditions (Fig. 6D). These results suggest that the inhibitory effect of physapubescin on VEGF secretion may depend on the expression of both HIF-1α and HIF-2α.

### Physapubescin inhibits expression of vimentin in 786-O cells and *in vivo* angiogenesis in the 786-O xenograft model

**Figure 7:**
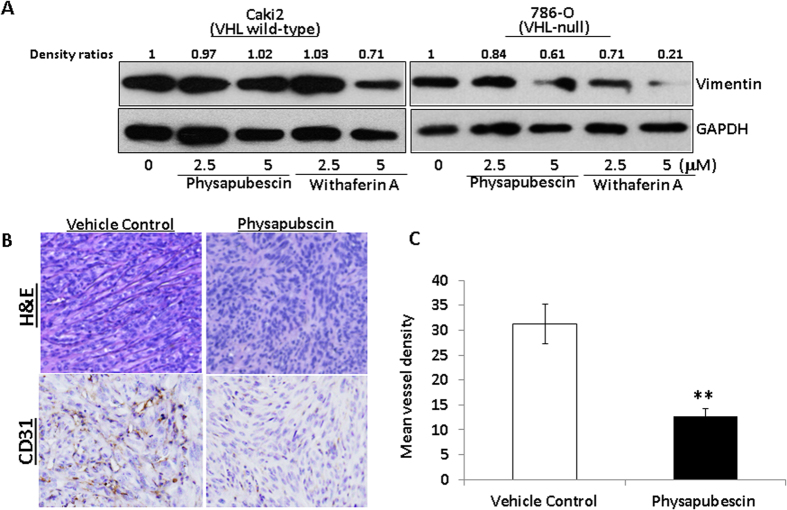
Physapubescin decreases the protein expression of vimentin in 786-O cells and inhibits *in vivo* angiogenesis in 786-O xenograft tumors. (**A**) The protein expression of vimentin at indicated treatments for 24 hours was analyzed by Western blotting. GAPDH was detected as a loading control. A representative blot was shown from three independent experiments. The protein levels were quantified by densitometry. Density ratios relative to GAPDH were shown on the top of each Western blotting band. (**B**) Immunostaining of CD31 protein in 786-O xenograft tumors. The mice bearing 786-O tumors were randomly divided, pair-matched into treatment and control groups of six mice each, and five days on and two days off dosing was begun with vehicle control or physapubescin at 30 mg/kg. At the end of the experiment; tumors were excised from each mouse, fixed in buffered formalin, embedded in paraffin blocks and tissue slides were prepared. Control immunostaining was performed with IgG isotype alone; Slides were counterstained with hematoxylin and photographed using a light microscope. Original magnification: X200. (**C**) CD31-positive cells were counted in 12 fields in each group. The percentage of CD31-positive cells was calculated as vessel density and presented as mean ± SEM. The mean vessel density is significantly lower in the physapubescin treatment group (n = 6) than that in the vehicle control group (n = 6). “*” and “**” denote “*P* < 0.05” and “*P* < 0.01” respectively.

Since there is a close relationship among hypoxia, angiogenesis and vimentin and vimentin is a direct target of withaferin A, a well-studied withanolide^11^, we examined the protein expression of vimentin in VHL wild-type Caki-2 cells and VHL null 786-O cells after physapubescin and withaferin A treatments. Figure 7A shows that both physapubescin and withaferin A decreased the protein expression of vimentin in 786-O cells in a dose-dependent manner. There is no change in the protein expression of vimentin in Caki-2 cells that were treated with up to 5 micromolar concentration of physapubescin for 24 hours (Fig. 7A). Physapubescin is less potent than withaferin A in reduction of vimentin protein expression (Fig. 7A).

Lastly, we examined the vivo anti-angiogenesis effect of physapubescin in the 786-O xenograft model. Hematoxylin and Eosin (H&E) staining showed that there was a significant enrichment in vascularization in the tumors of vehicle control treated mice compared to those treated with physapubescin in the 786-O xenograft tumors (Fig. 7B). Immunohistochemical analysis of tumor tissues further revealed that physapubescin significantly reduced mean vessel density as measured by CD31 staining by about 56%. Taken together, physapubescin demonstrated *in vivo* anti-angiogenesis in the VHL-null 786-O RCC xenograft model.

## Discussion

Conventional chemotherapeutic drugs unselectively kill rapidly proliferating cells in both normal and cancer tissues and they are often associated with severe toxicities and side effects. A new, intellectually appealing strategy for the development of next–generation cancer killing agents would be to exploit vulnerabilities that are associated with the genetic and epigenetic alterations in cancer cells^27^. ccRCC is uniquely suited for this exploitation since the majority (more than 80%) of ccRCC harbor VHL mutation, which conveys distinct characteristics on tumor cells and drives tumor development via HIF (either HIF-1α or HIF-2α) stabilization^27^,^29^,^30^.

Physapubescin belongs to the family of withanolides that contain an α, β-unsaturated ketone moiety in the A ring, which can react with protein thiol-nucleophiles and form Michael addition adducts^10^,^11^,^12^,^13^,^14^. Compared to other known withanolides, such as withaferin A isolated from *Withania somnifera*^6^,^15^,^16^,^17^,^18^,^31^, the biological activity of physapubescin remains largely unexplored. Here we are the first to report that physapubescin, a major withanolide from hairy ground-cherry, selectively induces apoptosis in VHL-null RCC cell lines via down-regulation of HIF -1/2α expression and up-regulation of CHOP and DR5. It also inhibits angiogenesis in the VHL-null 786-O xenograft model. A recently published study by Kim *et al*.^32^ showed that withaferin A did not affect VEGF production and HIF-1α stabilization induced by *H. pylori* in AGS cells. Here, we observed that physapubescin down-regulated the expression of HIF-1α and HIF-2α and decreased VEGF production in RCC cell lines. There are some obvious structural differences between physapubescin and withaferin A. Physapubescin has a nine-carbon epoxy-δ-lactol side chain at C-17 position and a 15-acetoxy substitution at C-15, whereas withaferin A shows an α, β-unsaturated δ-lactone side chain and an H group at C-15. Our results suggest that physapubescin may have a different structure-activity from withaferin A in terms of regulating HIF-1/2α expression and VEGF production in RCC cell lines.

Physapubescin increases both mRNA and protein expression of DR5 and activates caspase-8 and -3, leading to PARP cleavage and apoptotic morphology, such as cell rounding, nuclear fragmentation and condensation. These results indicate activation of the death receptor mediated apoptotic pathway by physapubescin. In addition, the up-regulation of DR5 expression was accompanied by increased expression of CHOP and decreased expression HIF-2α. CHOP is a transcriptional factor that regulates DR5 expression and both CHOP and DR5 are critically involved in endoplasmic reticulum stress-induced apoptosis^28^. Interestingly, the effect of physapubescin on expression of CHOP and DR5 was only observed in VHL deficient cells with HIF-2α over-expression, but not in VHL wild-type RCC cells that do not have HIF-2α expression. Although the DR5 promoter does not contain a HIF responsive element (5′G/ACGTG3′)^33^, HIF-2α, but not HIF-1α, has been shown to up-regulate DR5 expression from its promoter^34^. Therefore, it is plausible that HIF-2α and CHOP may form a complex at the DR5 promoter to regulate its transcription in RCC cells. VHL is required for degradation of HIF-2α, and development of VHL-deficient RCC has been shown to depend on HIF-2α activation^22^,^23^,^24^. These results suggest that HIF-2α may play an important role in the mechanism of physapubescin-induced apoptosis in VHL-deficient RCC cells.

**Figure 8:**
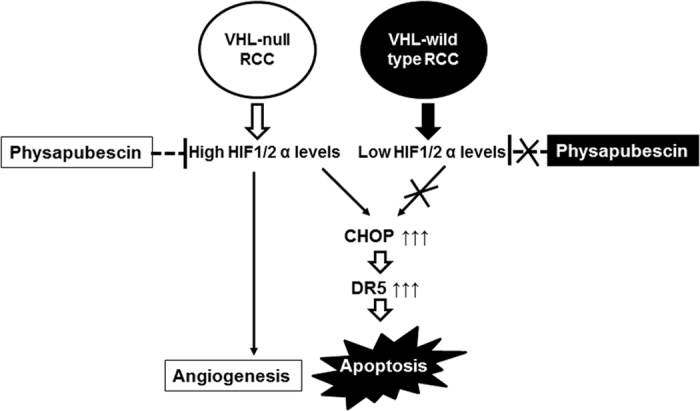
Model of a mechanism by which physapubescin down-regulates the expression of HIF 1/2α and selectively up-regulates the expression of CHOP and DR5 leading to apoptosis in VHL-null RCC cells but not in VHL wild-type RCC cells.

The model of a mechanism by which physapubescin selectively induces apoptosis in VHL-null RCC cells is summarized in Fig. 8. Physapubescin decreases the protein expression of HIF-1/2α and then causes elevated levels of CHOP, which transcriptionally activates DR5 leading to apoptosis in VHL-null RCC cells. In VHL wild type RCC cells, the relevant targets (i.e. HIF-1/2α) for physapubescin inducing apoptosis are absent under normoxia condition and thus physapubescin is not able to activate the CHOP/DR5 mediated apoptotic pathway. Further studies are underway to investigate mechanisms by which physapubescin regulates HIF-1/2α expression. Additionally, the inter-relationship among HIF-2α, CHOP and DR5 that are regulated by physapubescin remains largely unclear and warrants further investigation.

Hypoxic condition and cobalt chloride treatment significantly enhanced the expression of HIF-1α and HIF-2α and increased VEGF secretion. Interestingly, physapubescin decreased expression of HIF-1α and HIF-2α as well as VEGF secretion. The decreased protein levels of HIF-1α and HIF-2α by physapubescin under hypoxic condition and cobalt chloride treatment are associated with enhanced expression of DR5 and PARP cleavage. These results indicate that RCCs are more sensitive to physapubescin’s apoptotic effect under hypoxic conditions. We also demonstrated that physapubescin significantly decreased vessel density as measured by CD31 staining in tumor tissues. Withaferin A demonstrated anti-angiogenic effects both *in vitro* and *in vivo*^11^. Withaferin A has been shown to bind to vimentin and its anti-angiogenesis effect was attenuated in vimentin-deficient mice^11^. This result suggested that vimentin may be an important target for the anti-angiogenesis effect of withaferin A. We also found that physapubescin decreased the protein expression of vimentin in VHL-null 786-O cells but not in VHL wild-type Caki-2 cells (Fig. 7A). Treatment of 30 mg/kg physapubescin (i. p., on a five-day on and two-day off schedule for 5 weeks) resulted in reduction of angiogenesis in tumors. Therefore, the ability of physapubescin to down-regulate HIF-1/2α and vimentin levels led to anti-angiogenesis effects, which suggests that physapubescin may be particularly effective against highly vascularized RCC tumors.

TRAIL functions as a ligand to activate certain death receptors, including DR5, and induces apoptosis primarily in a variety of tumor cells, while remaining nontoxic to normal cells^35^. We further demonstrated that physapubescin acts synergistically with TRAIL for inducing apoptosis in VHL-null RCC cells but not in VHL wild-type Caki-2 cells. This effect of physapubescin may be due to its induction of DR5 expression only in VHL-null RCC cells but not in VHL wild-type Caki-2 cells. Contrary to our results, Lee *et al*.^18^ reported that withaferin A induced the expression of DR5 and sensitized TRAIL-induced apoptosis in VHL wild-type Caki cells. These results further suggest that physapubescin and withaferin A may have a differential up-stream target for regulating DR5 expression in RCC cells. Currently, clinical trials have been carried out to test TRAIL-based therapies in combination with chemotherapy or the proteasome inhibitor bortezomib compared with the respective standard of care therapy alone in patients with different types of cancers^36^,^37^,^38^,^39^,^40^,^41^. Although TRAIL-based therapies were well tolerated, the antitumor activity of these therapies has not achieved statistical significance in any of the reported clinical trials 36,37,38,39,40,41. In this regard, physapubescin may be further developed to work as a potent VHL-null ccRCC selective TRAIL sensitizer to be used in combination with TRAIL in the treatment of ccRCC.

## Materials and Methods

### Compounds

physapubescin (3.1 g) was isolated from the air-dried stems and leaves of *Physalis pubescens* L. (9.3 kg, collected from Shenyang, Liaoning Province, China) in our laboratory and was identified by comparing its ^1^H and ^13^C NMR spectroscopic data (supplementary Table 1 and supplementary Fig. 1A,B) with those reported in the literatures^2^. The purity was measured by HPLC [column: Aglient Zorbax SB-C18, 4.6 × 150 mm, 5 μm; solvent phase: methanol−H_2_O (60:40)] and determined to be 98.1% (supplementary Fig. 2). Withaferin A was purchased from Sigma Inc. (St Louis, MO). Physapubescin and withaferin A were dissolved in DMSO to make a stock solution, aliquoted and stored at −20 °C. The DMSO concentration was kept below 0.05% in all cell cultures used and did not exert any detectable effect on cell growth or death.

### Cell lines and Reagents

The 786-O, A-498, Caki-2, ACHN, cell lines were obtained from American Type Culture Collection (ATCC, Manassas, VA, USA). RCC4/pcDNA3 and RCC4/VHL cell lines were purchased from Sigma (St Louis, MO). All cell lines used in this study were within 20 passages after receipt. The cell lines were tested and authenticated by ATCC or Sigma. The 786-O cell line was cultured in RPMI-1640 medium supplemented with 10% fetal bovine serum (FBS). A-498 and ACHN cell lines were grown in EMEM medium supplemented with 10% FBS. Caki-2 cell line was cultured in a McCoy’s medium supplemented with 10% FBS. RCC4/pcDNA3 and RCC4/VHL cell lines were grown in DMEM medium supplemented with 10% FBS, 2 mM Glutamine, and 0.5 mg/ml G418. Antibodies for DR5, PARP, caspase-3, caspase-8 and CHOP were from Cell Signaling Technology (Danvers, MA), and HIF-2α antibody from GeneTex Inc. (Irvine, CA). Antibody for β-tubulin was from Santa Cruz Biotechnology, Inc. (Santa Cruz, CA). TRAIL Recombinant Protein was from Peprotech Inc. (Rocky Hill, NJ). Thymidine, 3-(4, 5-dimethylthiazol-2-yl)-2, 5-diphenyltetrazolium bromide (MTT) was obtained from Sigma (St Louis, MO).

### MTT assay

Cells were seeded onto 24 well plates at a density of 2 × 10^4^ cells for 24 hours (h) and then treated with physapubescin at indicated concentrations. In addition, 786-O and Caki-2 cells were treated by physapubescin with or without recombinant TRAIL protein added to each well to study the combined effects of physapubescin and TRAIL. After 72 h incubation, 200 μL of MTT solution was added to each well and incubated at 37°C for 2 h. The MTT solution was then aspirated and 200 μL of dissolving buffer was added to each well. Cell viability was determined by measuring absorbance at 570 nm in a microplate reader (Bio-Rad, Hercules, CA). Dose response curves were generated as a percentage of vehicle control treated cells using Excel software, and IC_50_ values were estimated graphically from the plot.

### Soft Agar Colony Formation

A total number of 5,000 786-O cells were seeded on the top layer containing 0.35% solidified agar in complete medium in 6-well plates and the bottom layer consisted of 0.8% agar in complete medium. Vehicle control (0.05% DMSO) or indicated concentrations of physapubescin in complete medium were added and replaced every 3 days. After 3 weeks of cell seeding, the number of colonies was counted under an inverted phase-contrast microscope at 100× magnification and a group of >10 cells was indicated as a colony.

### DAPI Nuclear Staining

786-O and Caki-2 cells (4 × 10^4^ cells/well) were cultured on chamber slides for 24 h. The cells were then treated with different concentrations of physapubescin for 24 h. After treatments, the cells were rinsed in 1× PBS for 3 times, and fixed in 4% paraformaldehyde. The fixed cells were mounted in Vector shield medium containing DAPI (Vector Laboratories, Inc., Burlingame, CA) in a dark place and visualized with a Nikon Eclipse TE2000-S (200× magnification) microscope under ultraviolet light. Apoptotic cells were identified by the nuclear condensation and fragmentation.

### Western Blotting Analysis

Volumes of clarified protein lysates containing equal amounts of protein (50 μg) were separated on 10–12% sodium deodecyl sulfate-polyacrylamide gel electrophoresis (SDS-PAGE) and electrophoretically transferred to Hybond-ECL membranes (GE Healthcare, Piscataway, NJ). The blots were then probed with primary antibody, followed by secondary antibodies as described previously^42^. Immunoreactive bands were visualized using an enhanced chemiluminescence detection system (Thermo Scientific, Rockford, IL).

### Caspase Activity Assay

The Caspase-Glo^®^ 3/7 and Caspase-Glo^®^ 8 Assays (Promega, Madison, WI) for measuring caspase-3/7 and caspase-8 activities in treated cells, respectively, were performed according to the manufacturer’s instructions. Briefly, 786-O cells were cultured on a 24-well plate and treated with 0.05% DMSO or physapubescin (1.25, 2.5 and 5 μmol/L) for 24 h. Then 100 μL of Caspase-Glo^®^ 3/7 or Caspase-Glo^®^ 8 reagents were added into each well and the luminescences of each sample, positive and negative controls were measured in a luminometer (GloMax^®^-MultiDetection System).

### Flow Cytometric Analysis of Apoptosis

Cells were treated with 0.05% DMSO or physapubescin at indicated concentrations for 24 h. After treatments, the cells were harvested, rinsed with PBS, and fixed in 70% ethanol at 4 °C for 18 h. After washing twice with PBS, the cells were stained with FITC-conjugated Annexin V and propidium iodide according to the manufacturer’s protocol (PharMingen)^43^. The analyses of cells were done by using appropriate scatter gates to exclude cellular aggregated and debris cells. Ten thousand events were counted for each sample.

### Quantitative Reverse Transcription-Polymerase Chain Reaction (RT-PCR)

Total RNA was isolated using the RNA bee method (TEL TEST Inc.) as described previously^43^,^44^. Real-time quantitative PCR amplification reactions for DR5 mRNA levels were carried out using the CFX96 Touch Real-Time PCR Detection System (Bio-Rad) as described previously^44^. The sequences of primers for DR5 and β-actin are available upon request. Data were analyzed by using the comparative Ct method, where Ct is the cycle number at which fluorescence first exceeds the threshold. The Ct values from each sample were obtained by subtracting the values for β-actin Ct from the target gene Ct value. The variation of β-actin Ct values is < 0.5 among different samples. A one cycle difference of Ct value represents a 2-fold difference in the level of mRNA. Specificity of resulting PCR products was confirmed by melting curves and agarose gel.

### VEGF ELISA assay

VEGF secretion was measured in RCC cells using Human VEGF ELISA kits (RayBiotech, Inc., Norcross, GA). Cells were plated in 6-well plates for 24 h, and then treated with physapubescin both under normoxia and hypoxia conditions for 24 h. Supernatants were collected and VEGF protein levels were determined by ELISA according to the manufacturer’s instructions. Absorbance was measured using a microplate reader (Bio-Rad, Hercules, CA, USA).

### *In Vivo* Antitumor Activity

NCR-nu/nu (nude) mice were obtained from Taconic (Germantown, NY). 786-O Cells were concentrated to 2 × 10^6^ per 200 μL and injected s.c. into the right flank of each mouse. Seven days later, the mice were randomly divided and pair matched into treatment and control groups of 6 mice each, and intraperitoneally injected with vehicle control or 30 mg physapubescin/kg body weight on a five-day on and two-day off schedule. The tumor sizes were measured every 3 days and calculated by the formula: 0.5236 x L1 x (L2)^2^, where L1 is the long axis and L2 is the short axis of the tumor. All of the animal studies were approved and carried out in accordance with guidelines set forth by the Institutional Animal Care and Use Committee at University of California (Irvine, CA).

### Histology and Immunohistochemistry

Tumor tissue slides were de-paraffinized and dehydrated using Slide Brite (Sasco Chemical Group, Inc.). Antigen was retrieved using 0.05 M Glycine-HCL buffer, pH 6.5, containing 0.01% (w/v) EDTA, at 95°C for 20 min and stained with an antibody against human CD31 (1:500). Staining was visualized with diaminobenzadine using the Cell and Tissue Staining kit (R&D Systems). The immunostaining was scored as positive or negative for CD31 by a pathologist.

### Statistical Analysis

Comparisons of cell density, number of colonies, relative levels of mRNA expression and relative levels of protein expression between the different transfections were conducted using Student’s t test. For tumor growth experiments, repeated-measures ANOVA was used to examine the differences in tumor sizes among different transfections, time points, and transfection-time interactions. Additional post-test was done to examine the differences in tumor sizes between vector control and other transfections at each time point by using conservative Bonferroni method. All statistical tests were two sided. P < 0.05 was considered statistically significant.

## Additional Information

**How to cite this article**: Chen, L. *et al*. Physapubescin selectively induces apoptosis in VHL-null renal cell carcinoma cells through down-regulation of HIF-2α and inhibits tumor growth. *Sci. Rep.* **6**, 32582; doi: 10.1038/srep32582 (2016).

## Supplementary Material

Supplementary Information
