# Supplementary material for: Physapubescin selectively induces apoptosis in VHL-null renal cell carcinoma cells through down-regulation of HIF-2α and inhibits tumor growth
Source: Sci Rep. 2016 Sep 1;6:32582. doi: 10.1038/srep32582 (PMC5007653; doi:10.1038/srep32582)
Supplement: Supplementary Information [file srep32582-s1.pdf]

Physapubescin selectively induces apoptosis in VHL-null renal cell carcinoma cells through down-regulation of HIF-2a and inhibits tumor growth

Lixia Chen,[†,‡] Guiyang Xia,[‡] Feng Qiu,[§] Chunli Wu,[†,‖] Andria P. Denmon,[†] XiaolinZi*,[†]

[†]*Departments of Urology and Pharmacology, Chao Family Comprehensive Cancer Center, University of California, Irvine, Orange, CA 92868, USA*

[‡]*Department of Natural Products Chemistry, School of Traditional Chinese Materia Medica, Key Laboratory of Structure-Based Drug Design & Discovery, Ministry of Education, Shenyang Pharmaceutical University, Shenyang 110016, People's Republic of China*

[§]*School of Chinese Materia Medica and Tianjin State Key Laboratory of Modern Chinese Medicine, Tianjin University of Traditional Chinese Medicine, Tianjin 300193, People's Republic of China*

[‖]*Co-innovation Center of Henan Province for New Drug R & D and Preclinical Safety and School of Pharmaceutical Sciences, Zhengzhou University, 100 Kexue Avenue, Zhengzhou, Henan 450001, China.*

[*]To whom all the correspondence should be addressed at Department of Urology, University of California, Irvine, 101 The City Drive South, Rt.81 Bldg.55 Rm.302, Orange CA 92868, U. S. A. Phone (714) 456-8316, Fax (714)456-1786, E-mail: **xzi@uci.edu**

Chen et al. supplementary Fig. 1

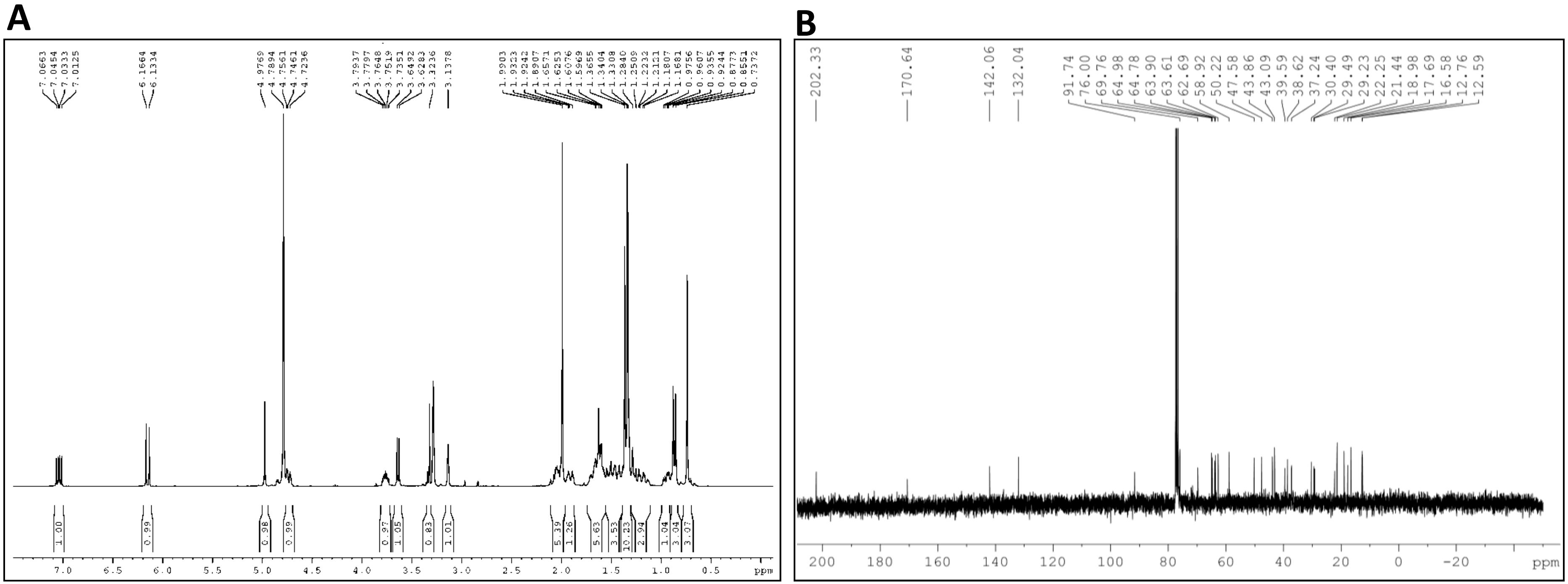

Supplementary Fig.1 Chemical characterization of physapubescin from *Physalis pubescens L.*. A, ^{1}H NMR spectrum (300 MHz, in CD_3OD) of physapubescin. **B,** ^{13}C NMR spectrum (75 MHz, in $CDCl_3$) of physapubescin.

Chen et al. supplementary Fig. 2

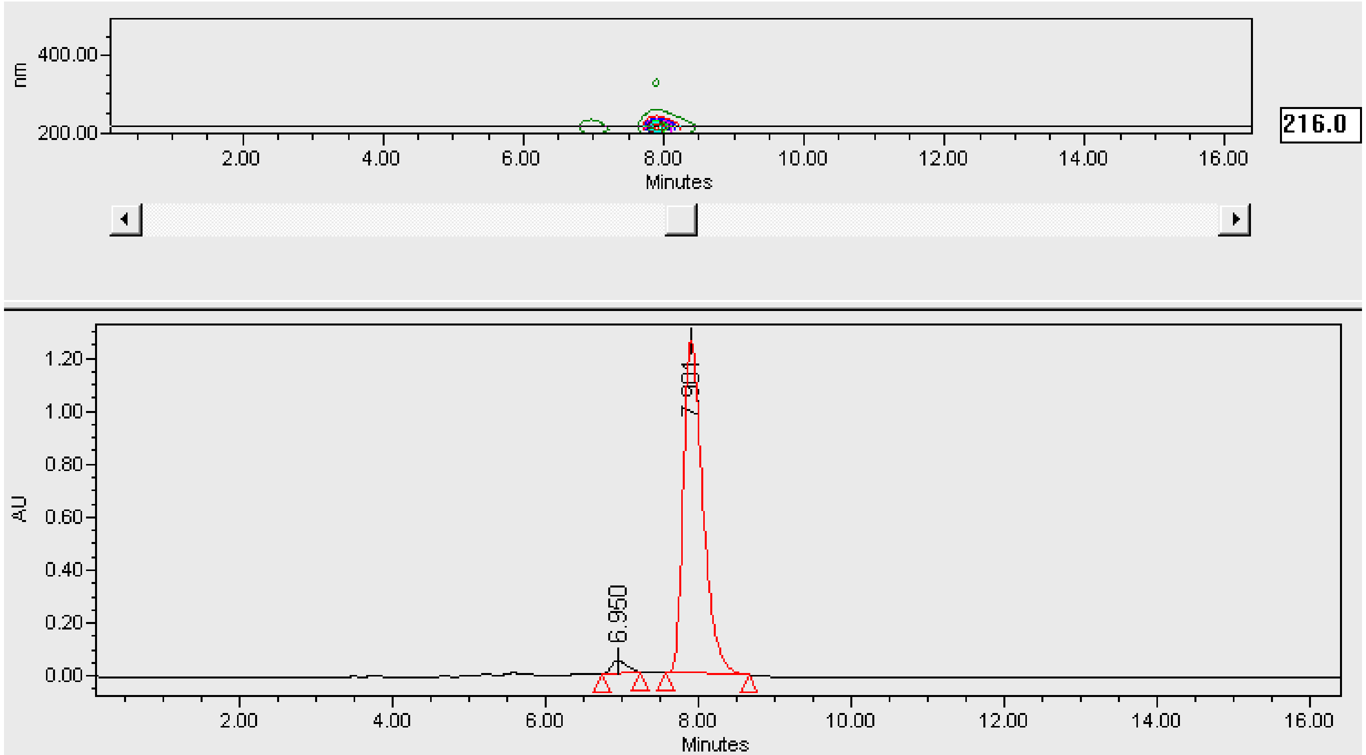

Supplementary Fig. 2 A representative chromatograph of physapubescin analyzed by HPLC [column: Aglient Zorbax SB-C18, 4.6 × 150 mm, 5 μm; solvent phase: methanol−H2O (60:40)].

Chen et al. supplementary Fig. 3

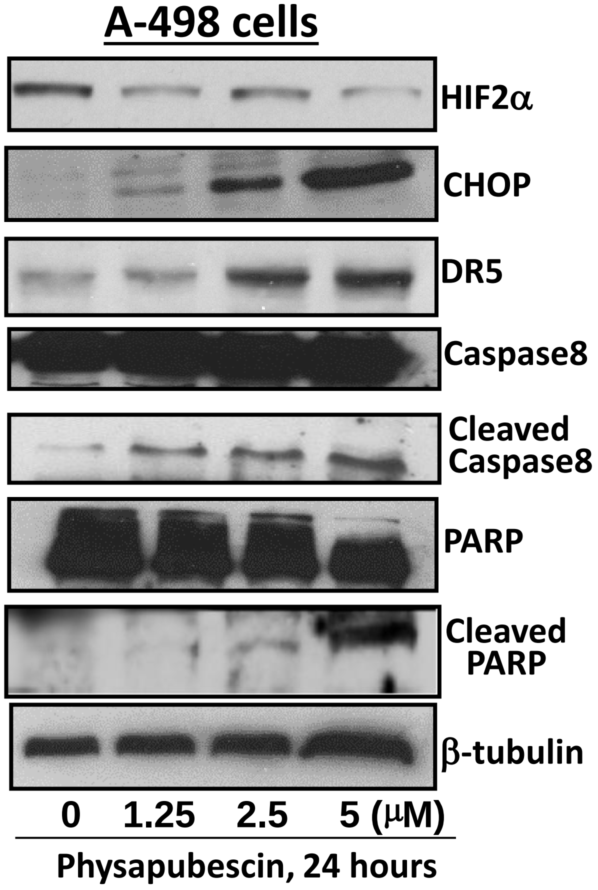

Supplementary Fig.3 Physapubescin decreases the expression of HIF-2α and increased the expression of CHOP and DR5 leading to activation of caspase cascade in VHL null A-498 cells. Protein expression of HIF-2α, CHOP, DR5, cleaved caspase-8 and PARP after indicated treatments hours was detected by Western blotting analysis. β-Tubulin was detected as a loading control. A representative blot was shown from three independent experiments.

Chen et al. supplementary Fig. 4

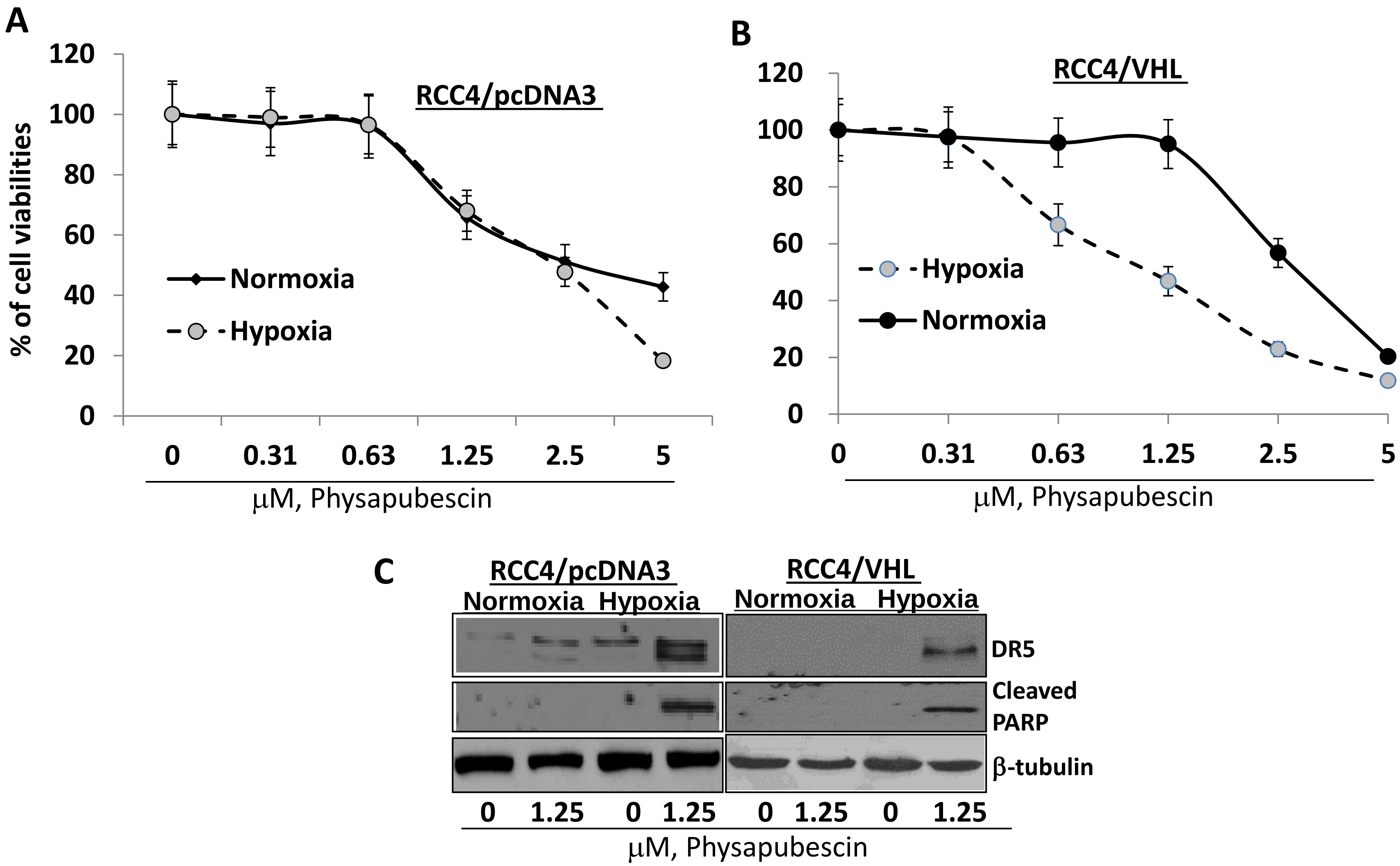

Supplementary Fig.4 The effects of physapubescin on reducing cell viabilities and modulating expression of related biomarkers are enhanced under hypoxia vs. normoxia conditions. RCC4/pcDNA3 cells were seeded at a density of 5×10^4 cells/well in six well plates under normoxic (21% O2), hypoxic (1% O2) conditions. After 24 hours of seeding, (A) cells were treated with 0.05% DMSO or physapubescin at the indicated concentrations for 72 hours. Cell densities were measured by MTT assay. Each value represents mean ± SEM of three samples for each treatment; (B) the protein expression of DR5 and cleaved PARP at indicated treatments for 24 hours was analyzed by Western blotting. β-Tubulin was detected as a loading control. A representative blot was shown from three independent experiments.
